# Poly[[tetra­aquatetrakis­[μ_3_-5-(pyridine-4-carboxamido)­isophthalato]cobalt(II)digadolinium(III)] tetra­hydrate]

**DOI:** 10.1107/S1600536811038074

**Published:** 2011-09-30

**Authors:** Yi-Fang Deng, Man-Sheng Chen, Chun-Hua Zhang, Dai-Zhi Kuang

**Affiliations:** a Key Laboratory of Functional Organometallic Materials, Hengyang Normal University, Department of Chemistry and Materials Science, Hengyang, Hunan 421008, People’s Republic of China

## Abstract

In the centrosymmetric polymeric title compound, {[CoGd_2_(C_14_H_8_N_2_O_5_)_4_(H_2_O)_4_]·4H_2_O}_*n*_, the Gd^III^ cation is coordinated by one water mol­ecule and four pyridine-4-carboxamido­isophthalate (*L*) anions in a distorted square-anti­prismatic arrangement, while the Co^II^ cation, located on an inversion center, is coordinated by two pyridyl-N atoms, two carboxyl­ate-O atoms and two water mol­ecules in a distorted octa­hedral geometry. The asymmetric unit contains two anionic *L* ligands: one bridges two Gd cations and one Co cation through two carboxyl groups and one pyridine-N atom; the other bridges two Gd cations and one Co cation through two carboxyl groups and the uncoordinated pyridine-N atom is hydrogen-bonded to the adjacent coordinated water mol­ecule. Extensive O—H⋯O and N—H⋯O hydrogen bonds are present in the crystal structure.

## Related literature

For related hetero-metallic complexes, see: Chen *et al.* (2011 ▶); Gu & Xue (2006 ▶); Liang *et al.* (2000 ▶); Prasad *et al.* (2007 ▶); Zhao *et al.* (2003 ▶, 2004 ▶). 
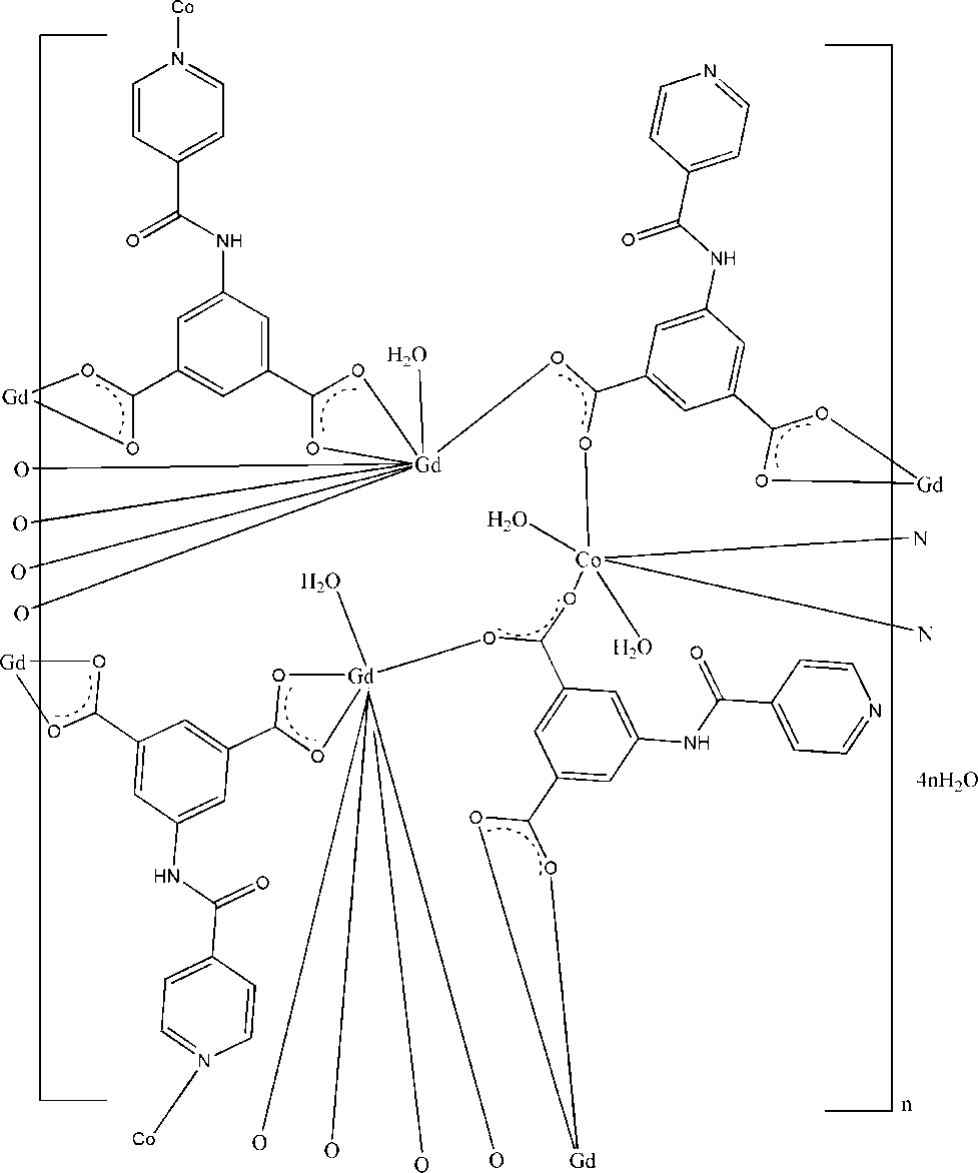


## Experimental

### 

#### Crystal data

[CoGd_2_(C_14_H_8_N_2_O_5_)_4_(H_2_O)_4_]·4H_2_O
*M* *_r_* = 1654.45
Triclinic, 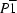

*a* = 10.1457 (14) Å
*b* = 10.8728 (15) Å
*c* = 13.7552 (19) Å
α = 79.123 (2)°
β = 78.844 (3)°
γ = 86.317 (2)°
*V* = 1461.3 (3) Å^3^
*Z* = 1
Mo *K*α radiation
μ = 2.62 mm^−1^
*T* = 293 K
0.20 × 0.16 × 0.10 mm

#### Data collection

Bruker SMART 1000 CCD area-detector diffractometer
Absorption correction: multi-scan (*SADABS*; Bruker, 2001 ▶) *T*_min_ = 0.622, *T*_max_ = 0.779
7307 measured reflections
5053 independent reflections
4462 reflections with *I* > 2σ(*I*)
*R*_int_ = 0.045

#### Refinement

*R*[*F* ^2^ > 2σ(*F* ^2^)] = 0.031
*wR*(*F* ^2^) = 0.070
*S* = 1.00
5053 reflections
430 parameters
1 restraint
H-atom parameters constrained
Δρ_max_ = 1.23 e Å^−3^
Δρ_min_ = −0.88 e Å^−3^

### 

Data collection: *SMART* (Bruker, 2007 ▶); cell refinement: *SAINT* (Bruker, 2007 ▶); data reduction: *SAINT*; program(s) used to solve structure: *SHELXTL* (Sheldrick, 2008 ▶); program(s) used to refine structure: *SHELXTL*; molecular graphics: *SHELXTL*; software used to prepare material for publication: *SHELXTL*.

## Supplementary Material

Crystal structure: contains datablock(s) global, I. DOI: 10.1107/S1600536811038074/xu5327sup1.cif

Structure factors: contains datablock(s) I. DOI: 10.1107/S1600536811038074/xu5327Isup2.hkl

Additional supplementary materials:  crystallographic information; 3D view; checkCIF report

## Figures and Tables

**Table 1:** Selected bond lengths (Å)

Co1—O1	2.083 (3)
Co1—O1*W*	2.178 (4)
Co1—N4^i^	2.159 (4)
Gd1—O2	2.246 (3)
Gd1—O2*W*	2.365 (3)
Gd1—O3^ii^	2.436 (3)
Gd1—O4^ii^	2.420 (3)
Gd1—O6	2.487 (3)
Gd1—O7	2.408 (3)
Gd1—O8^iii^	2.475 (3)
Gd1—O9^iii^	2.382 (3)

Symmetry codes: (i) 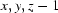; (ii) 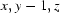; (iii) 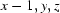.

**Table 2:** Hydrogen-bond geometry (Å, °)

*D*—H⋯*A*	*D*—H	H⋯*A*	*D*⋯*A*	*D*—H⋯*A*
N1—H1⋯O4*W*^iv^	0.86	2.16	2.999 (6)	166
N3—H3⋯O4^v^	0.86	2.15	2.942 (6)	152
O1*W*—H1*WA*⋯O6^vi^	0.82	2.25	2.992 (5)	151
O1*W*—H1*WB*⋯O3*W*^vii^	0.85	2.03	2.753 (6)	143
O2*W*—H2*WA*⋯O3*W*^viii^	0.85	2.40	3.130 (6)	144
O2*W*—H2*WB*⋯N2^i^	0.85	1.92	2.676 (6)	147
O3*W*—H3*WA*⋯O3^vii^	0.85	1.91	2.737 (6)	163
O3*W*—H3*WB*⋯O8^iii^	0.85	1.97	2.781 (6)	160
O4*W*—H4*WA*⋯O9^ix^	0.85	2.26	3.097 (6)	170
O4*W*—H4*WB*⋯O9^v^	0.85	2.18	3.028 (6)	172

Symmetry codes: (i) 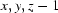; (iii) 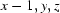; (iv) 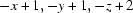; (v) 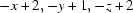; (vi) 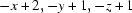; (vii) 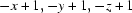; (viii) 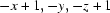; (ix) 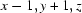.

**Fig. 1.**
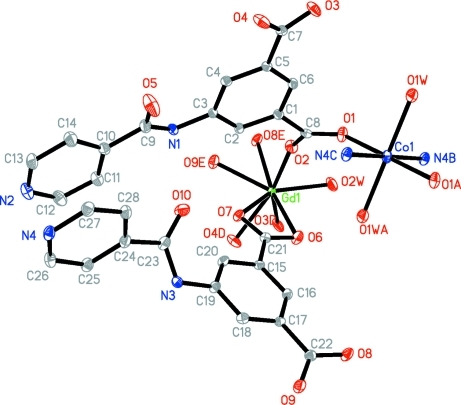
The ORTEP drawing of the title compound (I). Displacement ellipsoids are drawn at 30% probability level. [Symmetry codes: (i) 2 - x, 1 - y, 1 - z (ii) 2 - x, 1 - y, 2 - z (iii) x, y, -1 + z (iv) x,-1 + y, z (v)-1 + x, y, z.]

**Fig. 2.**
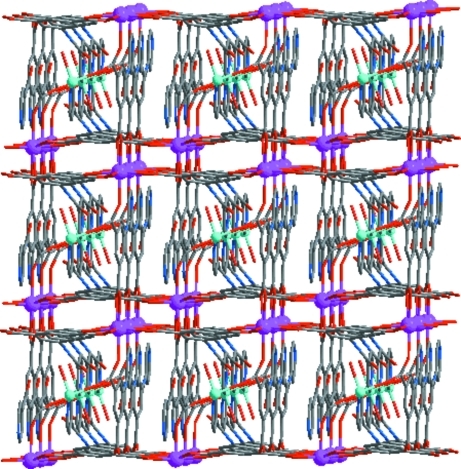
Projection showing the three-dimensional structure of the compound.

## supplementary crystallographic information

### Comment

The rational synthesis and investigation of 3d-4f or 4d-4f hetero-metallic complexes are challenge for chemists and have attracted increasing attention in last few years since the competitive reaction containing 3d-4f metal ions in conjunction with ligands often result in formation of a mixture of homometallic assemblies rather than hetero-metallic analogous (Liang *et al.*, 2000; Zhao *et al.*, 2003; Zhao *et al.*, 2004; Gu *et al.*, 2006; Prasad *et al.*, 2007). So we have recently prepared a new lanthanide(III)-transition metal(II) coordination polymer, [GdCo_0.5_(H_2_O)_2_(*L*)_2_]_n_.2nH_2_O, (I) through hydrothermal condition.

In the title compound, the central Gd^III^ ion is eight-coordinated by seven O atoms from four ligands and one water molecule, which forming a distorted square antiprismatic geometry(Fig. 1). It is interesting that the carboxyl groups of two unique *L*^2-^ ligands exhibit the different coordination modes: one coordinated to two Gd^III^ and one Co^II^ atoms using its two carboxylate groups with µ_1_-η^1^:η^1^-chelate and µ_2_-η^1^:η^1^-bis-monodentate coordination modes while the pyridyl group is free of coordination, the other one coordinated to two Gd^III^ through the carboxylate groups with µ_1_-η^1^:η^1^-chelate coordination mode and one Co^II^*via* the pyridyl group. Based on the coordination modes of the carboxylate and pyridyl groups of *L*^2-^ ligands, a complicated three-dimensional network is formed (Fig. 2), which is similar to the complex {[LnCo_0.5_(INAIP)_2_(H_2_O)_2_].2H_2_O}_n_ (Chen, *et al.* 2011).

### Experimental

A mixture of 0.05 mmol Gd(NO_3_)_3_.6H_2_O (21.8 mg. 0.05 mmol), H_2_L (28.6 mg, 0.1 mmol), Co(OAc)_2_.4H_2_O (13.1 mg, 0.05 mmol), NaOH (6.0 mg, 0.15 mmol), MeOH (4 ml) and H_2_O (6 ml) was heated in a 16 mL capacity Teflon-lined reaction vessel at 433 K for 4 days, the reaction mixture was cooled to room temperature over a period of 40 h. The product was collected by filtration, washed with H_2_O and air-dried.

### Refinement

H atoms bonded to C atoms were placed geometrically and refined as riding atoms. The pyridyl N atoms were found from a difference Fourier maps and refined as riding, with N—H = 0.86 Å, and the water H atoms were found from Fourier difference maps and refined with restraints for O—H distances (0.82–0.8515 Å) with *U*_iso_(H) = 1.2*U*_eq_(O).

### Crystal data

**Table:**

[CoGd_2_(C_14_H_8_N_2_O_5_)_4_(H_2_O)_4_]·4H_2_O	*Z* = 1
*M**_r_* = 1654.45	*F*(000) = 819
Triclinic, *P*1	*D*_x_ = 1.880 Mg m^−^^3^
Hall symbol: -P 1	Mo *K*α radiation, λ = 0.71073 Å
*a* = 10.1457 (14) Å	Cell parameters from 5652 reflections
*b* = 10.8728 (15) Å	θ = 2.7–28.2°
*c* = 13.7552 (19) Å	µ = 2.62 mm^−^^1^
α = 79.123 (2)°	*T* = 293 K
β = 78.844 (3)°	Block, pink
γ = 86.317 (2)°	0.20 × 0.16 × 0.10 mm
*V* = 1461.3 (3) Å^3^	

### Data collection

**Table:**

Bruker SMART 1000 CCD area-detector diffractometer	5053 independent reflections
Radiation source: fine-focus sealed tube	4462 reflections with *I* > 2σ(*I*)
graphite	*R*_int_ = 0.045
φ and ω scans	θ_max_ = 25.0°, θ_min_ = 2.1°
Absorption correction: multi-scan (*SADABS*; Bruker, 2001)	*h* = −12→10
*T*_min_ = 0.622, *T*_max_ = 0.779	*k* = −12→12
7307 measured reflections	*l* = −15→16

### Refinement

**Table:**

Refinement on *F*^2^	Primary atom site location: structure-invariant direct methods
Least-squares matrix: full	Secondary atom site location: difference Fourier map
*R*[*F*^2^ > 2σ(*F*^2^)] = 0.031	Hydrogen site location: inferred from neighbouring sites
*wR*(*F*^2^) = 0.070	H-atom parameters constrained
*S* = 1.00	*w* = 1/[σ^2^(*F*_o_^2^) + (0.0217*P*)^2^] where *P* = (*F*_o_^2^ + 2*F*_c_^2^)/3
5053 reflections	(Δ/σ)_max_ = 0.001
430 parameters	Δρ_max_ = 1.23 e Å^−^^3^
1 restraint	Δρ_min_ = −0.88 e Å^−^^3^

### Special details

**Table:**

Geometry. All e.s.d.'s (except the e.s.d. in the dihedral angle between two l.s. planes) are estimated using the full covariance matrix. The cell e.s.d.'s are taken into account individually in the estimation of e.s.d.'s in distances, angles and torsion angles; correlations between e.s.d.'s in cell parameters are only used when they are defined by crystal symmetry. An approximate (isotropic) treatment of cell e.s.d.'s is used for estimating e.s.d.'s involving l.s. planes.
Refinement. Refinement of *F*^2^ against ALL reflections. The weighted *R*-factor *wR* and goodness of fit *S* are based on *F*^2^, conventional *R*-factors *R* are based on *F*, with *F* set to zero for negative *F*^2^. The threshold expression of *F*^2^ > σ(*F*^2^) is used only for calculating *R*-factors(gt) *etc*. and is not relevant to the choice of reflections for refinement. *R*-factors based on *F*^2^ are statistically about twice as large as those based on *F*, and *R*- factors based on ALL data will be even larger.

### Fractional atomic coordinates and isotropic or equivalent isotropic displacement parameters (Å^2^)

**Table:**

	*x*	*y*	*z*	*U*_iso_*/*U*_eq_	
Co1	1.0000	0.5000	0.5000	0.0225 (2)	
Gd1	0.68073 (2)	0.07754 (2)	0.702891 (17)	0.01596 (9)	
C1	0.7021 (5)	0.4733 (4)	0.7262 (4)	0.0203 (11)	
C2	0.6789 (5)	0.4174 (5)	0.8277 (3)	0.0204 (11)	
H2	0.6736	0.3308	0.8458	0.024*	
C3	0.6639 (4)	0.4907 (4)	0.9019 (3)	0.0193 (11)	
C4	0.6653 (4)	0.6204 (4)	0.8743 (4)	0.0197 (11)	
H4	0.6539	0.6703	0.9235	0.024*	
C5	0.6838 (5)	0.6749 (4)	0.7734 (3)	0.0173 (11)	
C6	0.7035 (5)	0.6027 (4)	0.6993 (4)	0.0209 (11)	
H6	0.7176	0.6405	0.6317	0.025*	
C7	0.6865 (5)	0.8143 (4)	0.7469 (4)	0.0193 (11)	
C8	0.7333 (5)	0.3940 (5)	0.6454 (4)	0.0221 (11)	
C9	0.6653 (5)	0.4830 (5)	1.0810 (4)	0.0290 (13)	
C10	0.6520 (5)	0.4000 (5)	1.1831 (4)	0.0254 (12)	
C11	0.6510 (5)	0.2702 (5)	1.2015 (4)	0.0302 (13)	
H11	0.6536	0.2269	1.1489	0.036*	
C12	0.6462 (5)	0.2065 (5)	1.2977 (4)	0.0354 (14)	
H12	0.6463	0.1195	1.3083	0.042*	
C13	0.6398 (6)	0.3854 (6)	1.3606 (4)	0.0395 (15)	
H13	0.6347	0.4256	1.4152	0.047*	
C14	0.6451 (6)	0.4584 (6)	1.2657 (4)	0.0356 (14)	
H14	0.6442	0.5454	1.2574	0.043*	
C15	1.0257 (5)	0.1207 (4)	0.8319 (4)	0.0196 (11)	
C16	1.1577 (5)	0.1051 (4)	0.7839 (4)	0.0224 (11)	
H16	1.1755	0.0884	0.7186	0.027*	
C17	1.2619 (5)	0.1141 (5)	0.8325 (4)	0.0205 (11)	
C18	1.2342 (5)	0.1428 (4)	0.9300 (4)	0.0219 (11)	
H18	1.3043	0.1485	0.9634	0.026*	
C19	1.1033 (5)	0.1625 (5)	0.9764 (4)	0.0222 (11)	
C20	0.9992 (5)	0.1483 (5)	0.9280 (4)	0.0218 (11)	
H20	0.9107	0.1574	0.9603	0.026*	
C21	0.9089 (5)	0.1090 (4)	0.7833 (4)	0.0209 (11)	
C22	1.4062 (5)	0.0983 (5)	0.7846 (4)	0.0230 (12)	
C23	0.9939 (5)	0.2964 (5)	1.0919 (4)	0.0265 (12)	
C24	0.9909 (5)	0.3393 (5)	1.1902 (4)	0.0257 (12)	
C25	0.9824 (5)	0.2572 (5)	1.2814 (4)	0.0269 (12)	
H25	0.9817	0.1711	1.2846	0.032*	
C26	0.9752 (5)	0.3078 (5)	1.3671 (4)	0.0277 (13)	
H26	0.9670	0.2532	1.4286	0.033*	
C27	0.9848 (5)	0.5065 (5)	1.2779 (4)	0.0281 (13)	
H27	0.9861	0.5923	1.2761	0.034*	
C28	0.9885 (5)	0.4655 (5)	1.1898 (4)	0.0271 (12)	
H28	0.9895	0.5225	1.1301	0.033*	
N1	0.6472 (4)	0.4306 (4)	1.0036 (3)	0.0218 (10)	
H1	0.6233	0.3539	1.0173	0.026*	
N2	0.6415 (5)	0.2612 (5)	1.3771 (3)	0.0371 (12)	
N3	1.0794 (4)	0.2000 (4)	1.0720 (3)	0.0239 (10)	
H3	1.1204	0.1604	1.1183	0.029*	
N4	0.9793 (4)	0.4299 (4)	1.3671 (3)	0.0246 (10)	
O1	0.8077 (3)	0.4368 (3)	0.5644 (2)	0.0302 (9)	
O2	0.6813 (4)	0.2877 (3)	0.6641 (3)	0.0322 (9)	
O3	0.6714 (4)	0.8704 (3)	0.6616 (3)	0.0351 (10)	
O4	0.7066 (4)	0.8773 (3)	0.8103 (3)	0.0358 (10)	
O5	0.6911 (5)	0.5913 (4)	1.0738 (3)	0.0564 (13)	
O6	0.9267 (3)	0.0946 (3)	0.6916 (3)	0.0312 (9)	
O7	0.7925 (3)	0.1148 (3)	0.8335 (2)	0.0242 (8)	
O8	1.4396 (3)	0.0994 (4)	0.6915 (3)	0.0301 (9)	
O9	1.4954 (3)	0.0854 (4)	0.8378 (3)	0.0413 (11)	
O10	0.9241 (4)	0.3512 (4)	1.0332 (3)	0.0392 (10)	
O1W	0.9258 (4)	0.6827 (3)	0.4333 (3)	0.0329 (9)	
H1WB	0.8653	0.7196	0.4704	0.039*	
H1WA	0.9888	0.7243	0.3993	0.039*	
O2W	0.7218 (4)	0.0952 (3)	0.5257 (3)	0.0404 (10)	
H2WA	0.6782	0.0365	0.5148	0.048*	
H2WB	0.6868	0.1627	0.4982	0.048*	
O3W	0.3006 (4)	0.1662 (5)	0.5329 (3)	0.0747 (16)	
H3WA	0.3248	0.1472	0.4746	0.090*	
H3WB	0.3576	0.1389	0.5703	0.090*	
O4W	0.4138 (5)	0.8385 (4)	0.9854 (3)	0.0680 (15)	
H4WA	0.4328	0.9019	0.9390	0.082*	
H4WB	0.4384	0.8526	1.0379	0.082*	

### Atomic displacement parameters (Å^2^)

**Table:**

	*U*^11^	*U*^22^	*U*^33^	*U*^12^	*U*^13^	*U*^23^
Co1	0.0265 (6)	0.0248 (6)	0.0171 (5)	−0.0039 (4)	−0.0018 (4)	−0.0071 (4)
Gd1	0.01600 (14)	0.01569 (14)	0.01703 (14)	−0.00071 (9)	−0.00433 (10)	−0.00367 (9)
C1	0.021 (3)	0.020 (3)	0.020 (3)	−0.001 (2)	−0.004 (2)	−0.002 (2)
C2	0.021 (3)	0.017 (3)	0.020 (3)	−0.002 (2)	0.001 (2)	0.000 (2)
C3	0.013 (3)	0.025 (3)	0.018 (3)	0.001 (2)	−0.001 (2)	0.001 (2)
C4	0.016 (3)	0.022 (3)	0.022 (3)	0.001 (2)	−0.006 (2)	−0.004 (2)
C5	0.015 (3)	0.015 (3)	0.020 (3)	−0.003 (2)	−0.003 (2)	−0.001 (2)
C6	0.020 (3)	0.021 (3)	0.019 (3)	−0.002 (2)	−0.002 (2)	−0.001 (2)
C7	0.017 (3)	0.019 (3)	0.023 (3)	0.001 (2)	−0.006 (2)	−0.006 (2)
C8	0.021 (3)	0.020 (3)	0.025 (3)	0.003 (2)	−0.009 (2)	−0.002 (2)
C9	0.028 (3)	0.033 (3)	0.025 (3)	−0.002 (3)	−0.002 (2)	−0.002 (2)
C10	0.018 (3)	0.033 (3)	0.024 (3)	−0.002 (2)	−0.005 (2)	−0.001 (2)
C11	0.036 (3)	0.030 (3)	0.026 (3)	−0.006 (3)	−0.009 (3)	−0.001 (2)
C12	0.039 (4)	0.032 (3)	0.034 (3)	−0.011 (3)	−0.011 (3)	0.004 (3)
C13	0.047 (4)	0.054 (4)	0.020 (3)	0.004 (3)	−0.009 (3)	−0.012 (3)
C14	0.040 (4)	0.037 (4)	0.030 (3)	−0.002 (3)	−0.009 (3)	−0.004 (3)
C15	0.017 (3)	0.021 (3)	0.022 (3)	0.002 (2)	−0.006 (2)	−0.005 (2)
C16	0.024 (3)	0.026 (3)	0.017 (3)	0.001 (2)	−0.004 (2)	−0.006 (2)
C17	0.014 (3)	0.027 (3)	0.021 (3)	0.000 (2)	−0.005 (2)	−0.003 (2)
C18	0.018 (3)	0.025 (3)	0.026 (3)	−0.002 (2)	−0.010 (2)	−0.008 (2)
C19	0.021 (3)	0.026 (3)	0.022 (3)	−0.001 (2)	−0.003 (2)	−0.011 (2)
C20	0.017 (3)	0.026 (3)	0.024 (3)	0.001 (2)	−0.003 (2)	−0.010 (2)
C21	0.023 (3)	0.018 (3)	0.024 (3)	−0.001 (2)	−0.007 (2)	−0.007 (2)
C22	0.024 (3)	0.020 (3)	0.026 (3)	0.000 (2)	−0.005 (2)	−0.004 (2)
C23	0.021 (3)	0.035 (3)	0.026 (3)	−0.004 (2)	−0.003 (2)	−0.013 (3)
C24	0.016 (3)	0.042 (4)	0.022 (3)	0.000 (2)	−0.004 (2)	−0.011 (2)
C25	0.026 (3)	0.034 (3)	0.024 (3)	−0.001 (2)	−0.006 (2)	−0.012 (2)
C26	0.029 (3)	0.032 (3)	0.021 (3)	−0.009 (2)	−0.003 (2)	−0.002 (2)
C27	0.029 (3)	0.027 (3)	0.028 (3)	−0.003 (2)	−0.002 (3)	−0.007 (2)
C28	0.031 (3)	0.033 (3)	0.018 (3)	0.001 (2)	−0.005 (2)	−0.006 (2)
N1	0.025 (2)	0.018 (2)	0.019 (2)	−0.0009 (18)	−0.0032 (19)	0.0015 (18)
N2	0.043 (3)	0.042 (3)	0.026 (3)	−0.009 (2)	−0.010 (2)	0.003 (2)
N3	0.022 (2)	0.033 (3)	0.017 (2)	0.0036 (19)	−0.0085 (19)	−0.0049 (19)
N4	0.021 (2)	0.035 (3)	0.020 (2)	−0.004 (2)	−0.0025 (19)	−0.010 (2)
O1	0.030 (2)	0.039 (2)	0.021 (2)	−0.0097 (18)	0.0009 (17)	−0.0097 (17)
O2	0.048 (2)	0.016 (2)	0.033 (2)	−0.0053 (17)	−0.0041 (19)	−0.0062 (16)
O3	0.070 (3)	0.016 (2)	0.022 (2)	−0.0008 (18)	−0.015 (2)	−0.0015 (15)
O4	0.068 (3)	0.018 (2)	0.030 (2)	0.0048 (18)	−0.028 (2)	−0.0074 (16)
O5	0.113 (4)	0.028 (3)	0.034 (2)	−0.024 (3)	−0.023 (3)	−0.0009 (19)
O6	0.021 (2)	0.052 (3)	0.026 (2)	−0.0032 (17)	−0.0058 (17)	−0.0170 (18)
O7	0.0140 (19)	0.036 (2)	0.026 (2)	−0.0013 (15)	−0.0043 (16)	−0.0138 (16)
O8	0.0164 (19)	0.054 (3)	0.024 (2)	0.0013 (17)	−0.0044 (16)	−0.0168 (18)
O9	0.015 (2)	0.087 (3)	0.022 (2)	0.005 (2)	−0.0068 (17)	−0.010 (2)
O10	0.039 (2)	0.054 (3)	0.032 (2)	0.021 (2)	−0.016 (2)	−0.023 (2)
O1W	0.036 (2)	0.034 (2)	0.026 (2)	0.0018 (17)	0.0009 (17)	−0.0072 (17)
O2W	0.066 (3)	0.031 (2)	0.024 (2)	−0.005 (2)	−0.013 (2)	0.0016 (17)
O3W	0.060 (3)	0.132 (5)	0.031 (3)	0.028 (3)	−0.015 (2)	−0.014 (3)
O4W	0.139 (5)	0.037 (3)	0.030 (2)	−0.031 (3)	−0.015 (3)	−0.003 (2)

### Geometric parameters (Å, °)

**Table:**

Co1—O1^i^	2.083 (3)	C15—C16	1.389 (6)
Co1—O1	2.083 (3)	C15—C21	1.492 (6)
Co1—O1W	2.178 (4)	C16—C17	1.374 (6)
Co1—O1W^i^	2.178 (4)	C16—H16	0.9300
Co1—N4^ii^	2.159 (4)	C17—C18	1.406 (6)
Co1—N4^iii^	2.159 (4)	C17—C22	1.498 (7)
Gd1—O2	2.246 (3)	C18—C19	1.380 (6)
Gd1—O2W	2.365 (3)	C18—H18	0.9300
Gd1—O3^iv^	2.436 (3)	C19—C20	1.384 (7)
Gd1—O4^iv^	2.420 (3)	C19—N3	1.421 (6)
Gd1—O6	2.487 (3)	C20—H20	0.9300
Gd1—O7	2.408 (3)	C21—O7	1.252 (5)
Gd1—O8^v^	2.475 (3)	C21—O6	1.277 (5)
Gd1—O9^v^	2.382 (3)	C22—O9	1.256 (6)
C1—C6	1.386 (6)	C22—O8	1.257 (6)
C1—C2	1.395 (6)	C23—O10	1.223 (6)
C1—C8	1.505 (7)	C23—N3	1.352 (6)
C2—C3	1.389 (7)	C23—C24	1.506 (7)
C2—H2	0.9300	C24—C28	1.369 (7)
C3—C4	1.390 (6)	C24—C25	1.387 (7)
C3—N1	1.410 (6)	C25—C26	1.380 (7)
C4—C5	1.385 (6)	C25—H25	0.9300
C4—H4	0.9300	C26—N4	1.331 (6)
C5—C6	1.378 (7)	C26—H26	0.9300
C5—C7	1.491 (6)	C27—N4	1.340 (6)
C6—H6	0.9300	C27—C28	1.361 (7)
C7—O3	1.250 (6)	C27—H27	0.9300
C7—O4	1.257 (5)	C28—H28	0.9300
C8—O1	1.246 (5)	N1—H1	0.8600
C8—O2	1.263 (6)	N3—H3	0.8600
C9—O5	1.204 (6)	N4—Co1^vi^	2.159 (4)
C9—N1	1.345 (6)	O3—Gd1^vii^	2.436 (3)
C9—C10	1.507 (7)	O4—Gd1^vii^	2.420 (3)
C10—C11	1.387 (7)	O8—Gd1^viii^	2.475 (3)
C10—C14	1.390 (7)	O9—Gd1^viii^	2.382 (3)
C11—C12	1.365 (7)	O1W—H1WB	0.8501
C11—H11	0.9300	O1W—H1WA	0.8200
C12—N2	1.331 (7)	O2W—H2WA	0.8500
C12—H12	0.9300	O2W—H2WB	0.8499
C13—N2	1.326 (7)	O3W—H3WA	0.8515
C13—C14	1.387 (7)	O3W—H3WB	0.8500
C13—H13	0.9300	O4W—H4WA	0.8501
C14—H14	0.9300	O4W—H4WB	0.8501
C15—C20	1.382 (6)		
			
O1^i^—Co1—O1	180.00 (19)	O5—C9—C10	118.3 (5)
O1^i^—Co1—N4^ii^	87.68 (14)	N1—C9—C10	117.4 (5)
O1—Co1—N4^ii^	92.32 (14)	C11—C10—C14	117.2 (5)
O1^i^—Co1—N4^iii^	92.32 (14)	C11—C10—C9	125.6 (5)
O1—Co1—N4^iii^	87.68 (14)	C14—C10—C9	117.2 (5)
N4^ii^—Co1—N4^iii^	180.000 (1)	C12—C11—C10	119.3 (5)
O1^i^—Co1—O1W	86.75 (14)	C12—C11—H11	120.3
O1—Co1—O1W	93.25 (14)	C10—C11—H11	120.3
N4^ii^—Co1—O1W	90.98 (15)	N2—C12—C11	124.1 (5)
N4^iii^—Co1—O1W	89.02 (15)	N2—C12—H12	117.9
O1^i^—Co1—O1W^i^	93.25 (14)	C11—C12—H12	117.9
O1—Co1—O1W^i^	86.75 (14)	N2—C13—C14	123.2 (5)
N4^ii^—Co1—O1W^i^	89.02 (15)	N2—C13—H13	118.4
N4^iii^—Co1—O1W^i^	90.98 (15)	C14—C13—H13	118.4
O1W—Co1—O1W^i^	180.000 (1)	C13—C14—C10	119.2 (6)
O2—Gd1—O2W	82.58 (12)	C13—C14—H14	120.4
O2—Gd1—O9^v^	91.01 (14)	C10—C14—H14	120.4
O2W—Gd1—O9^v^	138.94 (13)	C20—C15—C16	119.9 (4)
O2—Gd1—O7	81.95 (12)	C20—C15—C21	117.8 (4)
O2W—Gd1—O7	139.26 (12)	C16—C15—C21	122.2 (4)
O9^v^—Gd1—O7	78.76 (11)	C17—C16—C15	120.2 (4)
O2—Gd1—O4^iv^	154.21 (12)	C17—C16—H16	119.9
O2W—Gd1—O4^iv^	120.60 (12)	C15—C16—H16	119.9
O9^v^—Gd1—O4^iv^	78.54 (13)	C16—C17—C18	119.5 (4)
O7—Gd1—O4^iv^	72.97 (12)	C16—C17—C22	122.6 (4)
O2—Gd1—O3^iv^	152.78 (12)	C18—C17—C22	117.9 (4)
O2W—Gd1—O3^iv^	71.09 (12)	C19—C18—C17	120.3 (4)
O9^v^—Gd1—O3^iv^	104.19 (13)	C19—C18—H18	119.9
O7—Gd1—O3^iv^	122.72 (12)	C17—C18—H18	119.9
O4^iv^—Gd1—O3^iv^	52.79 (11)	C18—C19—C20	119.4 (4)
O2—Gd1—O8^v^	85.71 (13)	C18—C19—N3	118.8 (4)
O2W—Gd1—O8^v^	85.98 (12)	C20—C19—N3	121.8 (4)
O9^v^—Gd1—O8^v^	53.03 (11)	C15—C20—C19	120.6 (4)
O7—Gd1—O8^v^	129.89 (11)	C15—C20—H20	119.7
O4^iv^—Gd1—O8^v^	105.71 (13)	C19—C20—H20	119.7
O3^iv^—Gd1—O8^v^	85.72 (13)	O7—C21—O6	120.2 (4)
O2—Gd1—O6	84.41 (13)	O7—C21—C15	119.0 (4)
O2W—Gd1—O6	87.95 (12)	O6—C21—C15	120.8 (4)
O9^v^—Gd1—O6	131.92 (11)	O9—C22—O8	119.4 (5)
O7—Gd1—O6	53.19 (11)	O9—C22—C17	119.6 (4)
O4^iv^—Gd1—O6	85.28 (13)	O8—C22—C17	121.0 (5)
O3^iv^—Gd1—O6	100.97 (13)	O9—C22—Gd1^viii^	57.6 (3)
O8^v^—Gd1—O6	169.00 (12)	O8—C22—Gd1^viii^	61.8 (3)
O2—Gd1—C22^v^	87.78 (13)	C17—C22—Gd1^viii^	176.5 (3)
O2W—Gd1—C22^v^	112.54 (14)	O10—C23—N3	123.3 (5)
O9^v^—Gd1—C22^v^	26.43 (13)	O10—C23—C24	119.8 (5)
O7—Gd1—C22^v^	104.25 (13)	N3—C23—C24	116.9 (5)
O4^iv^—Gd1—C22^v^	92.60 (14)	C28—C24—C25	118.8 (5)
O3^iv^—Gd1—C22^v^	95.88 (14)	C28—C24—C23	118.2 (5)
O8^v^—Gd1—C22^v^	26.61 (12)	C25—C24—C23	122.9 (5)
O6—Gd1—C22^v^	156.94 (13)	C24—C25—C26	117.7 (5)
O2—Gd1—C7^iv^	178.34 (13)	C24—C25—H25	121.2
O2W—Gd1—C7^iv^	96.19 (13)	C26—C25—H25	121.2
O9^v^—Gd1—C7^iv^	90.65 (14)	N4—C26—C25	124.0 (5)
O7—Gd1—C7^iv^	98.34 (13)	N4—C26—H26	118.0
O4^iv^—Gd1—C7^iv^	26.48 (12)	C25—C26—H26	118.0
O3^iv^—Gd1—C7^iv^	26.34 (12)	N4—C27—C28	123.6 (5)
O8^v^—Gd1—C7^iv^	95.32 (13)	N4—C27—H27	118.2
O6—Gd1—C7^iv^	94.44 (13)	C28—C27—H27	118.2
C22^v^—Gd1—C7^iv^	93.72 (14)	C27—C28—C24	119.2 (5)
C6—C1—C2	119.9 (4)	C27—C28—H28	120.4
C6—C1—C8	119.6 (4)	C24—C28—H28	120.4
C2—C1—C8	120.5 (4)	C9—N1—C3	125.7 (4)
C3—C2—C1	120.2 (5)	C9—N1—H1	117.2
C3—C2—H2	119.9	C3—N1—H1	117.2
C1—C2—H2	119.9	C12—N2—C13	117.0 (5)
C2—C3—C4	119.5 (4)	C23—N3—C19	121.6 (4)
C2—C3—N1	118.7 (4)	C23—N3—H3	119.2
C4—C3—N1	121.8 (4)	C19—N3—H3	119.2
C5—C4—C3	119.7 (4)	C26—N4—C27	116.6 (4)
C5—C4—H4	120.2	C26—N4—Co1^vi^	121.8 (3)
C3—C4—H4	120.2	C27—N4—Co1^vi^	121.3 (3)
C6—C5—C4	121.2 (4)	C8—O1—Co1	144.0 (3)
C6—C5—C7	120.8 (4)	C8—O2—Gd1	155.3 (3)
C4—C5—C7	118.0 (4)	C7—O3—Gd1^vii^	93.8 (3)
C5—C6—C1	119.5 (5)	C7—O4—Gd1^vii^	94.4 (3)
C5—C6—H6	120.3	C21—O6—Gd1	91.1 (3)
C1—C6—H6	120.3	C21—O7—Gd1	95.4 (3)
O3—C7—O4	118.8 (4)	C22—O8—Gd1^viii^	91.5 (3)
O3—C7—C5	121.0 (4)	C22—O9—Gd1^viii^	96.0 (3)
O4—C7—C5	120.1 (4)	Co1—O1W—H1WB	117.3
O3—C7—Gd1^vii^	59.9 (2)	Co1—O1W—H1WA	109.9
O4—C7—Gd1^vii^	59.1 (3)	H1WB—O1W—H1WA	117.8
C5—C7—Gd1^vii^	177.5 (3)	Gd1—O2W—H2WA	105.0
O1—C8—O2	123.9 (5)	Gd1—O2W—H2WB	110.8
O1—C8—C1	118.6 (4)	H2WA—O2W—H2WB	105.6
O2—C8—C1	117.5 (4)	H3WA—O3W—H3WB	111.2
O5—C9—N1	124.3 (5)	H4WA—O4W—H4WB	107.9
			
C6—C1—C2—C3	−3.2 (7)	O5—C9—N1—C3	4.4 (9)
C8—C1—C2—C3	173.3 (4)	C10—C9—N1—C3	−175.7 (4)
C1—C2—C3—C4	3.2 (7)	C2—C3—N1—C9	162.1 (5)
C1—C2—C3—N1	−176.9 (4)	C4—C3—N1—C9	−18.1 (7)
C2—C3—C4—C5	−1.0 (7)	C11—C12—N2—C13	0.8 (9)
N1—C3—C4—C5	179.2 (4)	C14—C13—N2—C12	−1.2 (9)
C3—C4—C5—C6	−1.3 (7)	O10—C23—N3—C19	5.8 (8)
C3—C4—C5—C7	−179.2 (4)	C24—C23—N3—C19	−171.8 (4)
C4—C5—C6—C1	1.3 (7)	C18—C19—N3—C23	132.4 (5)
C7—C5—C6—C1	179.2 (4)	C20—C19—N3—C23	−45.9 (7)
C2—C1—C6—C5	0.9 (7)	C25—C26—N4—C27	3.1 (7)
C8—C1—C6—C5	−175.7 (4)	C25—C26—N4—Co1^vi^	−169.8 (4)
C6—C5—C7—O3	18.9 (7)	C28—C27—N4—C26	−1.1 (8)
C4—C5—C7—O3	−163.2 (5)	C28—C27—N4—Co1^vi^	171.8 (4)
C6—C5—C7—O4	−159.9 (5)	O2—C8—O1—Co1	−118.9 (5)
C4—C5—C7—O4	18.0 (7)	C1—C8—O1—Co1	61.6 (7)
C6—C1—C8—O1	27.7 (7)	N4^ii^—Co1—O1—C8	−31.5 (6)
C2—C1—C8—O1	−148.9 (5)	N4^iii^—Co1—O1—C8	148.5 (6)
C6—C1—C8—O2	−151.9 (5)	O1W—Co1—O1—C8	−122.6 (6)
C2—C1—C8—O2	31.5 (7)	O1W^i^—Co1—O1—C8	57.4 (6)
O5—C9—C10—C11	−165.6 (5)	O1—C8—O2—Gd1	75.0 (10)
N1—C9—C10—C11	14.5 (8)	C1—C8—O2—Gd1	−105.4 (8)
O5—C9—C10—C14	11.8 (8)	O2W—Gd1—O2—C8	−92.6 (8)
N1—C9—C10—C14	−168.1 (5)	O9^v^—Gd1—O2—C8	128.1 (8)
C14—C10—C11—C12	−1.3 (8)	O7—Gd1—O2—C8	49.6 (8)
C9—C10—C11—C12	176.1 (5)	O4^iv^—Gd1—O2—C8	62.9 (9)
C10—C11—C12—N2	0.4 (9)	O3^iv^—Gd1—O2—C8	−107.1 (8)
N2—C13—C14—C10	0.4 (9)	O8^v^—Gd1—O2—C8	−179.1 (8)
C11—C10—C14—C13	0.9 (8)	O6—Gd1—O2—C8	−4.0 (8)
C9—C10—C14—C13	−176.7 (5)	C22^v^—Gd1—O2—C8	154.3 (8)
C20—C15—C16—C17	1.5 (7)	O4—C7—O3—Gd1^vii^	−4.0 (5)
C21—C15—C16—C17	−178.6 (5)	C5—C7—O3—Gd1^vii^	177.2 (4)
C15—C16—C17—C18	−1.8 (7)	O3—C7—O4—Gd1^vii^	4.0 (5)
C15—C16—C17—C22	−179.9 (4)	C5—C7—O4—Gd1^vii^	−177.2 (4)
C16—C17—C18—C19	−0.4 (7)	O7—C21—O6—Gd1	−3.4 (5)
C22—C17—C18—C19	177.8 (4)	C15—C21—O6—Gd1	177.4 (4)
C17—C18—C19—C20	2.8 (7)	O2—Gd1—O6—C21	86.0 (3)
C17—C18—C19—N3	−175.6 (4)	O2W—Gd1—O6—C21	168.7 (3)
C16—C15—C20—C19	1.0 (7)	O9^v^—Gd1—O6—C21	−0.3 (4)
C21—C15—C20—C19	−178.9 (5)	O7—Gd1—O6—C21	1.9 (3)
C18—C19—C20—C15	−3.2 (8)	O4^iv^—Gd1—O6—C21	−70.3 (3)
N3—C19—C20—C15	175.2 (4)	O3^iv^—Gd1—O6—C21	−121.0 (3)
C20—C15—C21—O7	−5.5 (7)	O8^v^—Gd1—O6—C21	112.2 (6)
C16—C15—C21—O7	174.6 (4)	C22^v^—Gd1—O6—C21	15.2 (5)
C20—C15—C21—O6	173.8 (4)	C7^iv^—Gd1—O6—C21	−95.2 (3)
C16—C15—C21—O6	−6.1 (7)	O6—C21—O7—Gd1	3.5 (5)
C16—C17—C22—O9	−167.4 (5)	C15—C21—O7—Gd1	−177.2 (4)
C18—C17—C22—O9	14.5 (7)	O2—Gd1—O7—C21	−91.0 (3)
C16—C17—C22—O8	13.5 (7)	O2W—Gd1—O7—C21	−22.3 (4)
C18—C17—C22—O8	−164.7 (5)	O9^v^—Gd1—O7—C21	176.4 (3)
O10—C23—C24—C28	−41.3 (7)	O4^iv^—Gd1—O7—C21	95.1 (3)
N3—C23—C24—C28	136.4 (5)	O3^iv^—Gd1—O7—C21	76.6 (3)
O10—C23—C24—C25	134.8 (6)	O8^v^—Gd1—O7—C21	−168.4 (3)
N3—C23—C24—C25	−47.5 (7)	O6—Gd1—O7—C21	−1.9 (3)
C28—C24—C25—C26	−1.5 (7)	C22^v^—Gd1—O7—C21	−176.6 (3)
C23—C24—C25—C26	−177.5 (5)	C7^iv^—Gd1—O7—C21	87.4 (3)
C24—C25—C26—N4	−1.8 (8)	O9—C22—O8—Gd1^viii^	−1.6 (5)
N4—C27—C28—C24	−2.0 (8)	C17—C22—O8—Gd1^viii^	177.6 (4)
C25—C24—C28—C27	3.3 (8)	O8—C22—O9—Gd1^viii^	1.6 (5)
C23—C24—C28—C27	179.5 (5)	C17—C22—O9—Gd1^viii^	−177.5 (4)

Symmetry codes: (i) −*x*+2, −*y*+1, −*z*+1; (ii) −*x*+2, −*y*+1, −*z*+2; (iii) *x*, *y*, *z*−1; (iv) *x*, *y*−1, *z*; (v) *x*−1, *y*, *z*; (vi) *x*, *y*, *z*+1; (vii) *x*, *y*+1, *z*; (viii) *x*+1, *y*, *z*.

### Hydrogen-bond geometry (Å, °)

**Table:**

*D*—H···*A*	*D*—H	H···*A*	*D*···*A*	*D*—H···*A*
N1—H1···O4W^ix^	0.86	2.16	2.999 (6)	166
N3—H3···O4^ii^	0.86	2.15	2.942 (6)	152
O1W—H1WA···O6^i^	0.82	2.25	2.992 (5)	151
O1W—H1WB···O3W^x^	0.85	2.03	2.753 (6)	143
O2W—H2WA···O3W^xi^	0.85	2.40	3.130 (6)	144
O2W—H2WB···N2^iii^	0.85	1.92	2.676 (6)	147
O3W—H3WA···O3^x^	0.85	1.91	2.737 (6)	163
O3W—H3WB···O8^v^	0.85	1.97	2.781 (6)	160
O4W—H4WA···O9^xii^	0.85	2.26	3.097 (6)	170
O4W—H4WB···O9^ii^	0.85	2.18	3.028 (6)	172

Symmetry codes: (ix) −*x*+1, −*y*+1, −*z*+2; (ii) −*x*+2, −*y*+1, −*z*+2; (i) −*x*+2, −*y*+1, −*z*+1; (x) −*x*+1, −*y*+1, −*z*+1; (xi) −*x*+1, −*y*, −*z*+1; (iii) *x*, *y*, *z*−1; (v) *x*−1, *y*, *z*; (xii) *x*−1, *y*+1, *z*.
